# Years of Life Lost of Patients With Early Adenocarcinoma of the Esophagogastric Junction Compared to Esophageal or Gastric Adenocarcinoma. A Registry‐Based Study

**DOI:** 10.1002/cam4.72079

**Published:** 2026-07-01

**Authors:** Elfriede Bollschweiler, Arnulf H. Hölscher, Brigitte Schumacher, Oliver Pech, Uta Drebber, in Cooperation with the German Cancer Registry, RKI

**Affiliations:** ^1^ Medical Faculty University of Cologne Cologne Germany; ^2^ Center for Esophageal Diseases Elisabeth‐Krankenhaus Essen Essen Germany; ^3^ Klinik für Gastroenterologie, Hepatologie und Transplantationsmedizin Universitätsmedizin Essen Essen Germany; ^4^ Klinik für Gastroenterologie und Interventionelle Endoskopie Krankenhaus Barmherzige Brüder Regensburg Regensburg Germany; ^5^ Institute of Pathology University of Cologne Cologne Germany

**Keywords:** early cancer, esophageal cancer, gastric cancer, prognosis, UICC‐TNM classification, upper gastrointestinal cancer, years of life lost

## Abstract

**Objective:**

This registry‐based study investigates patients with resected esophageal (EAC), cardiac (CAC), and gastric (GAC) pT1 adenocarcinoma (AC) concerning epidemiological, tumor‐related, and prognostic factors, analyzing especially the impact on survival and years of life lost (YLL).

**Background:**

As CAC is classified as esophageal cancer in the 7th and more specified in the 8th edition of the UICC‐TNM classification, it remains unclear if significant differences exist for pT1 CAC in comparison to EAC and GAC.

**Methods:**

We used the ICD‐O‐3 code comparing tumors in the esophagus (C15.0‐C15.9), cardia (C16.0), and stomach (C16.1‐C16.9). A total of 246,653 patients with these tumors were documented between 2010–2020 in the German Cancer Registry. Only ACs with histologically confirmed tumor infiltration into mucosa or submucosa (pT1) were included. YLL were calculated using life expectancy data from Germany.

**Results:**

The study population consists of 9.442 patients with resected pT1 AC. Age and sex differed significantly between CAC (*n* = 1.870) and EAC (*n* = 2.018) as well as between CAC and GAC (*n* = 5.554). Tumor‐related factors were significantly different between the three locations. Multivariate analysis showed significantly worse prognosis for CAC compared to the other locations. The median of YLL was 10.7 years for EAC, 8.5 years for CAC and 6.8 years for GAC (*p* < 0.0001).

**Conclusion:**

Patients with pT1 AC of the cardia differ from patients with EAC or GAC in terms of demographic, tumor‐related factors, prognosis, and YLL. Therefore, separate classification of tumors originating in the cardia is useful. Future studies should investigate the influence of therapy on prognosis and YLL.

## Graphical abstract

Notched Box‐and‐Whisker plots of years of life lost (YLL) comparing patients who died during the follow‐up with pT1 adenocarcinoma of the gastroesophageal junction. Patients with adenocarcinoma of the esophagus (EAC), cardia (CAC) or stomach (GAC) confined to the mucosa or submucosa lose a median of 10.7, 8.5 and 6.8 years of life, respectively, compared to the normal population, even though the tumors were treated at an early stage.

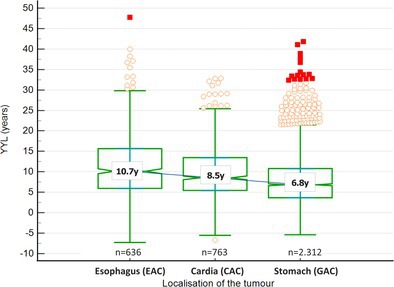

## 1 Introduction

Cancers in the upper gastrointestinal (UGI) tract are among the most common and deadly tumors globally [1]. The incidence rates have changed greatly during the last few decades, with divergent trends depending on histology and subsite. For esophageal cancer, the histological type “squamous cell carcinoma” dominates globally [2], but its incidence has decreased in western populations [3, 4]. Meanwhile, the incidence of adenocarcinoma (AC), the other main histological type of esophageal cancer, has increased rapidly in most western countries [5, 6]. Various causes are discussed, such as a high‐calorie diet resulting in obesity and the associated secondary diseases [7]. This contrasts with gastric cancer, which has shown a decreasing incidence in many Western countries over the same period [8]. Possible causes for this are also the change in dietary habits, such as a less salty diet, but also the treatment of *Helicobacter pylori* , which is known to be a proven risk factor for the development of gastric cancer [9].

Tumors—mostly adenocarcinoma—that develop in the cardia, the transition between the esophagus and the stomach are of special interest. Some authors assumed that these tumors have distinct malignant features compared with other esophageal and gastric cancers [10].

In western regions, the adenocarcinoma of the cardia is classified according to Siewert et al. [11], while the Nishi classification [12] is used in eastern countries. However, it can be difficult to determine the exact location of the tumor, depending on whether it is determined endoscopically or postoperatively [13]. It should be understood, for example, that the International Code for Classification of Diseases for Oncology Version 3 (ICD‐O‐3 code) separates tumors between the esophagus and stomach at the histopathological border of the Z‐line. Tumors described in the cardia, esophagogastric junction, esophagus, and stomach, ostium cardiacum, esophageal‐gastric junction are to be classified as gastric carcinomas with a separate subgroup with the ICD code C16.0. The classification rules for tumors in the cardia have changed in the UICC‐TNM Classifications of Malignant Tumors during the last 20 years. Tumors classified with the ICD‐Code C16.0 should be staged according to the rules of gastric cancer in the 6th edition and to the rules of esophageal cancer in the 7th edition [14, 15]. In contrast, the 8th and the 9th edition of the UICC‐TNM Classification categorized only cancers whose epicenter is within the proximal 2 cm of the cardia (Siewert Type I/II) as esophageal cancer [16, 17].

Based on the available studies, the question arises whether cardiac carcinoma is a tumor that is more likely to be assigned to the esophagus or stomach due to its origin, or whether tumors in this location require a separate category.

This question can traditionally be answered by analyzing epidemiological factors such as the patient's age and gender. Further criteria are the tumor characteristics, which are uniformly described using the Tumor, Nodes and Metastasis (TNM) classification and the therapies carried out. Success is measured by the patient's prognosis. Another way of assessing this is the impact of the tumor on life expectancy. “Burden of disease” is a concept developed in the 1990s by the Harvard School of Public Health, the World Bank, and the World Health Organization (WHO) to characterize death and loss of health due to diseases [18]. This concept is applied globally to map the health status of populations in a comprehensive and comparable manner according to a standardized concept [19]. The loss of years of life is therefore an additional way to assess the burden of a tumor disease. The years of life lost (YLL) are measured in comparison to the standard population.

An earlier study showed that patients with resected pT1 adenocarcinoma of the esophagus or stomach live a median of 8 years less than the age‐ and sex‐matched population [20]. There were no significant differences between tumors in the stomach or esophagus. The results are based on a small number of patients from a highly specialized surgical center, but very well investigated factors. Therefore, we want to examine whether this result can also be reproduced in a large number of patients and different supply structures. For this purpose, we used data from the German Cancer Registry, in which epidemiological and tumor‐related data as well as the date of death are documented nationwide.

The main aim of this study is to investigate how the epidemiological and tumor‐related factors of patients suffering from adenocarcinoma of the UGI‐tract differ between the various localizations—esophagus, cardia, and stomach. Patients with histologically confirmed adenocarcinomas confined to the mucosa or submucosa were included in order to minimize the influence of therapy on the outcome.

## 2 Material and Methods

### 2.1 Study Design and Data

The Center of Cancer Registry in Germany (ZfKD) located by the Robert Koch‐Institute has since 2010 registered the epidemiologic factors of all malignancies in Germany based on the data from the regional cancer registries. The data were available from 2010 until 2020 with a follow‐up until 2022 [21]. The tumors were classified according to the International Classification system for Diseases of Oncology Version 3 (ICD‐O‐3), using revision 10 [22].

For the present analysis we used the complete data set of the ZfKD with the ICD‐Code for topography C15 (esophagus) and C16 (stomach) including all subitems (C15.0–C15.9 and C16.0–C16.9). The type of histology was documented using ICD‐O‐3 (inclusive 1. Revision from 2013). For the analysis only malignant primary tumors classified as dignity/3 were selected. The tumor stage was documented using the UICC‐TNM‐Classification of Malignant Tumors version 6 and 7 [14, 15].

The following factors of the database were available and analyzed in this study: sex, age at diagnosis, year of birth, status alive or dead, year of death, tumor localization, histology of the tumor, tumor stage (T‐category), existence of lymph node metastasis (N‐category), grading of the tumor.

**FIGURE 1:**
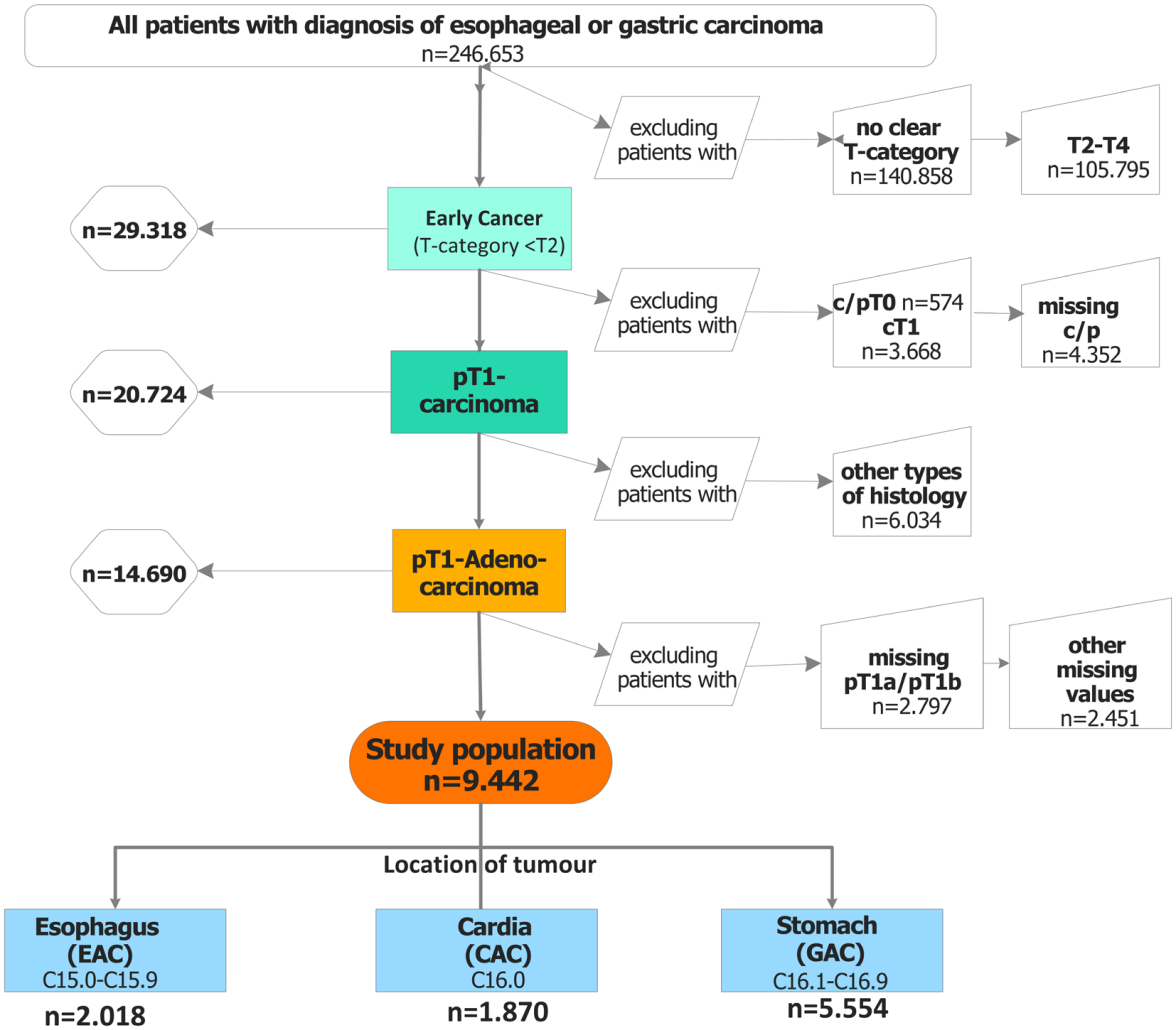
Workflow for selection of patients included in this study. Data from German Cancer Registry for patients with carcinoma of the esophagus or stomach (ICD‐O‐3: C15.0–C15.9 and C16.0–C16.9) documented between 2010 and 2020.

A total of 246,653 patients with the diagnosis of esophageal or gastric cancer were documented during the period from 2010 until 2020 in the German Cancer Registry. Figure 1 shows the flow‐chart of selected patients for this study. Early Cancer defined as T‐category less than T2 were 29,318; advanced categories (T2–T4) were documented in 105,795 cases. In this study, only cases with pT‐category based on results of histopathologic examination after endoscopic or surgical resection were analyzed. Therefore, cases with only clinical T‐category or missing T‐category were excluded.

To minimize the risk including patients categorized as early cancer after preoperative chemo‐ and/or radiotherapy, all cases classified as T0 or T1 without additional information were excluded.

The 20,724 pT1 carcinoma of the esophagus or stomach was further subdivided into different types of histology: 1948 squamous cell carcinoma (SCC), 898 neuroendocrine tumor, 14,690 adenocarcinoma, 1922 Signet‐ring cell carcinoma, and 1266 other types of histology.

The classification of the 14.690 adenocarcinomas included the following ICD‐0‐3 Codes: 8140, 8143, 8144, 8145, 8201, 8211, 8213, 8255, 8260, 8261, 8263, 8480.

In order to analyze as heterogeneous a patient population as possible, 2930 cases with missing values in other factors examined, such as N‐category or grading, were removed from the analysis. Furthermore, pT1 adenocarcinomas without subgrouping into mucosa (pT1a) or submucosa (pT1b) were not included in the evaluation (Figure 1).

The study population consists of 9442 patients with early AC of the esophagus or stomach.

We compared tumors localized in the esophagus (ICD‐O‐3 = C15.0–C15.9) = EAC, in the cardia (ICD‐O‐3 = 16.0) = CAC, and in the stomach (ICD‐O‐3 = C16.1–C16.9) = GAC.

The median follow‐up for all living patients was 6.5 years, min = 2 years, max = 12 years.

### 2.2 Statistics

Descriptive analysis included the frequency of nominal parameters, the median with lower (LQ) and upper (UQ) quartiles for numeric variables (ordinal or asymmetric distribution), and the mean for numeric variables with normal distribution. Univariate analysis for nominal variables was calculated using Chi‐squared statistics. The Kruskal–Wallis test was used to test whether the YLL differed between tumor locations. The multivariate analysis was performed using multiple regression analysis.

Univariate survival analysis was conducted according to the Kaplan–Meier method, and survival curves were compared with the log rank test. Multivariate analysis was performed with the Cox‐regression method. Significant differences between groups were defined with a *p <* 0.05.

To evaluate the number of years of life a person loses as consequences of dying early (YLL), we documented the number of expected years of life (EYL) for each patient. The EYL calculation was based on data from the Federal Statistical Office of Germany (Destatis) GENESIS V3.1.2020 [23]. The EYL was calculated for the date of diagnosis according to the age and sex of each patient and then registered. We used the average life expectancy (period life table) by years, sex, and completed age based on the year 2020. Details of the calculation of this estimation are described in GENESIS [23]. For patients dying during the follow‐up period, YLL was calculated as the difference between the expected time of death and the observed survival (OYS): YLL = EYL–OYS.

Age at diagnosis refers to the patient's age at initial diagnosis as reported by the treating physician. In principle, however, it cannot be ruled out that some patients may have received prior treatment, of which we are unaware.

For the selection of the adequate cases from the total data set and the statistic procedures the statistic program NCSS 2024 Statistical Software 2024 (NCSS LLC. Kaysville, Utah, USA, ncss.com/software/ncss) was used. The statistics program MedCalc Statistical Software version 23.2.1 (MedCalc Software Ltd., Ostend, Belgium; https://www.medcalc.org; 2025) was used for statistical graphics.

### 2.3 Ethics Approval

The use of the data set to answer scientific questions was reviewed and approved by the Ethics Committee of the Medical Faculty of the University of Cologne. Furthermore, the Robert Koch Institute approved the use of the data set in accordance with §8 of the Federal Cancer Registry Act.

## 3 Results

A total of 9442 patients with pT1 AC of the UGI who were reported to the Cancer Registry in Germany between 2010 and 2020 were available for the analysis. Table 1 shows the demographic and tumor‐related data broken down by tumor location. We compared EAC (*n* = 2018) with CAC (*n* = 1870) and GAC (*n* = 5554). The demographic factors differed significantly between tumors in the esophagus and cardia as well as between tumors in the cardia and stomach. Men were most commonly affected by AC with 85.0% of EAC, 80.6% of CAC and 61.0% of GAC. The average age at diagnosis was 66.8 years for esophageal, 69.2 years for cardia and 72.8 years for gastric carcinoma (Table 1).

**TABLE 1:** Demographics and tumor‐related factors for patients with pT1 adenocarcinoma of the gastroesophageal junction comparing tumors located in the esophagus, cardia and stomach.

	Esophagus EAC		E vs C *p*	Cardia CAC		C vs St *p*	Stomach GAC		All cases	
	*n*	%		*n*	%		*n*	%	*n*	%
*n*	2.018	100%		1.870	100%		5.554	100%	9.442	100%
Sex			0.0003			< 0.0001				*p* < 0.0001
Male	1.715	85.0%		1.508	80.6%		3.339	61.0%	6.613	70.0%
Female	303	15.0%		362	19.4%		2.164	39.0%	2.829	30.0%
Age										*p* < 0.001
Mean ± SD	66.84 ± 10.7 years			69.17 ± 10.9 years			72.78 ± 10.7 years			
Age group			< 0.0001			< 0.0001				*p* < 0.0001
< 50 years	120	5.9%		82	4.4%		209	3.8%	411	4.4%
50–59 years	447	22.2%		307	16.4%		553	10.0%	1.307	13.8%
60–69 years	633	31.4%		510	27.3%		1.123	20.2%	2.266	24.0%
≥ 70 years	818	40.5%		971	51.9%		3.669	66.1%	5.458	57.8%
pT‐category			< 0.0001			0.876				*p* < 0.0001
pT1a	1.218	60.4%		817	43.7%		2.438	43.9%	4.473	47.4%
pT1b	800	39.6%		1.053	56.3%		3.116	56.1%	4.969	52.6%
N‐category			0.0888			0.076				*p* = 0.0004
N0	1.828	90.6%		1.663	88.9%		4.853	87.4%	8.344	88.4%
N+	190	9.4%		207	11.1%		701	12.6%	1.098	11.6%
Grading			< 0.0001			< 0.0001				*p* < 0.0001
High Grade	637	31.6%		372	19.9%		1.168	21.0%	2.177	23.1%
Median Grade	1.012	50.1%		1.074	57.4%		2.478	44.6%	4.564	48.3%
Low Grade	369	18.3%		424	22.7%		1.908	34.4%	2.701	28.6%

There were significantly more often mucosal carcinomas in the EAC group compared to CAC, but there was no difference between CAC and GAC. The frequency of lymph node metastasis (LNM) was not different between the three tumor locations (Table 1).

### 3.1 Prognosis

The 5‐year survival rate (5y‐SR) for all patients with pT1 AC of the UGI‐tract was 69.0%. For patients with EAC the rate was 74.5% and significantly higher compared to those with CAC with 67.0% or GAC with a rate of 67.8% (*p* < 0.0001) (Table 2). All factors examined in the study showed a significant influence on survival in the univariate analysis (Table 2). At 74.6%, the 5y‐SR for patients with a tumor confined to the mucosa was more than 10% higher than for patients with submucosal carcinoma (64.0%). The survival rate for patients with LNM was significantly lower with 53.3% 5y‐SR compared to N0 patients with 71.1% 5y‐SR.

**TABLE 2:** Uni‐ and multivariate analysis of prognosis for patients with pT1 adenocarcinoma of the gastroesophageal junction.

Factor	*n*	5 year survival rate	Hazard ratio	Hazard ratio Cox‐regression	Significance
*n*	9.442	69.0%			*p* < 0.0001
Sex		*p* = 0.0079			
Male	6.613	68.1%	1	1	
Female	2.829	71.2%	0.909	0.909	*p* < 0.0001
Age group		*p* < 0.0001			
< 50 years	411	90.5%	1	1.	
50–59 years	1.307	85.0%	1.760	0.755	*p* < 0.0001
60–69 years	2.266	79.3%	2.726	1.161	*p* = 0.0029
≥ 70 years	5.458	59.5%	6.183	2.681	*p* < 0.0001
Location		*p* < 0.0001			
Esophagus	2.018	74.5%	1	1	
Cardia	1.870	67.0%	1.337	1.037	*p* = 0.0006
Stomach	5.554	67.8%	1.299	0.946	*p* = 0.0220
pT‐category		*p* < 0.0001			
pT1a	4.473	74.6%	1	1	
pT1b	4.969	64.0%	1.370	1.105	*p* < 0.0001
N‐category		*p* < 0.0001			
N0	8.344	71.1%	1	1	
N+	1.098	53.3%	1.856	1.228	*p* < 0.0001
Grading		*p* < 0.0001			
High Grade	2.177	73.6%	1	1	
Median Grade	4.564	68.1%	1.208	1.009	*p* = 0.669
Low Grade	2.701	67.0%	1.203	1.035	*p* = 0.189

**FIGURE 2:**
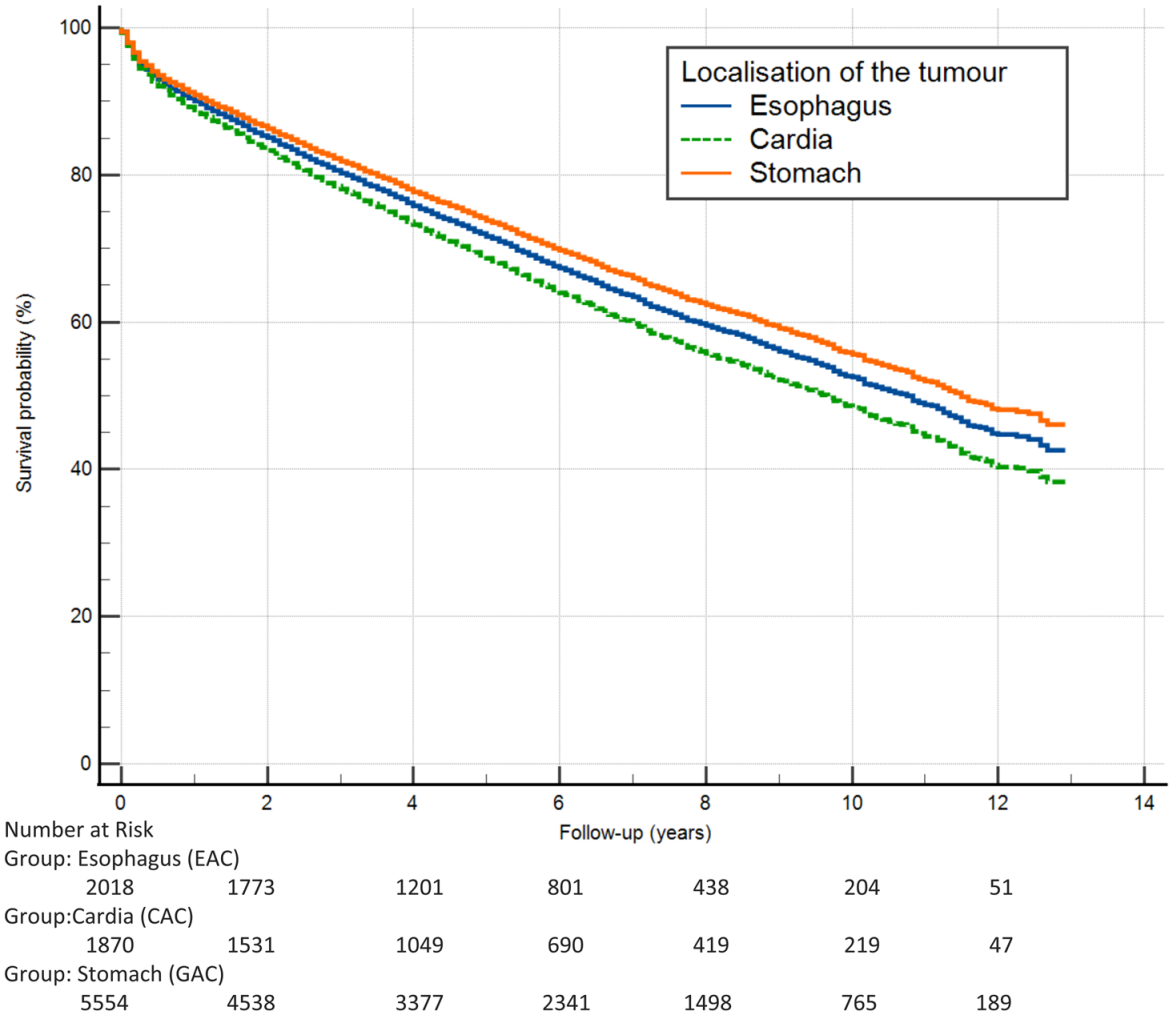
Multivariate survival curves including the factors age at diagnosis, sex, depth of tumor infiltration, existence of lymph node metastasis, and grading of the tumor comparing patients with pT1 adenocarcinoma of the esophagus (EAC), cardia (CAC), and stomach (GAC).

In the multivariate analysis, all factors investigated except grading had a significant influence on prognosis (Table 2). In the multivariate Cox regression analysis, the prognosis for CAC is significantly worse compared to the other two localizations. Figure 2 shows the survival curves for the different tumor locations, considering the mean values for the other factors included.

### 3.2 Years of Life Lost (YLL)

For the calculation of years of life lost, only patients who died during the follow‐up were included in the analysis. Of the 9442 patients in this study, 3711 deaths (39.3%) were documented in the registry. The frequency of death varied between tumor sites with the lowest rate of 31.5% in the esophagus, increasing to 40.8% for CAC and 41.6% for GAC (Table 3). The median number of YLL for patients who died during the study period was 7.5 years, with a maximum of 47.8 years and the lowest value of −7.3 years. A negative value means that patients lived longer than the age‐ and sex‐adjusted life expectancy.

**TABLE 3:** Uni‐ and multivariate analysis of years of life lost (YLL) for patients who died during the follow‐up with pT1 adenocarcinoma of the gastroesophageal junction.

Factor	All patients	Patients died during follow‐up			YYL**−**Median [LQ**−**UQ]		Multiple regression equitation	
	*n*	*n*	%	*p*	Years	*p*	Coefficient	*p*
*n*	9.442	3.711	39.3%		7.5 [7.3‐7.7]		Intercept = 32.2	*p* < 0.0001
Sex				*p* = 0.089		*p* < 0.006		
Male	6.613	2.636	39.9%		7.8 [4.5‐12.3]		1	
Female	2.829	1.075	38.0%		7.2 [3.9‐11.8]		1.063	*p* < 0.0001
Age group				*p* < 0.0001		*p* < 0.0001		
< 50 years	411	48	11.7%		31.8 [29.3‐34.3]		1	
50–59 years	1.307	244	18.7%		22.6 [19.9‐24.9]		−9.749	*p* < 0.0001
60–69 years	2.266	615	27.1%		14.9 [12.1‐24.6]		−17.674	*p* < 0.0001
≥ 70 years	5.458	2.804	51.4%		6.1 [3.4‐ 8.8]		−26.101	*p* < 0.0001
Location				*p* < 0.0001		*p* < 0.0001		
Esophagus	2.018	636	31.5%		10.7 [5.9‐15.6]		1	
Cardia	1.870	763	40.8%		8.5 [5.5‐13.5]		−0.171	*p* = 0.418
Stomach	5.554	2.312	41.6%		6.8 [3.7‐ 10.8]		−0.981	*p* < 0.0001
pT‐category				*p* < 0.0001		*p* = 0.0121		
pT1a	4.473	1.524	34.7%		7.3 [3.9‐11.9]		1	
pT1b	4.969	2.187	44.0%		7.8 [4.5‐12.3]		0.321	*p* = 0.0221
N‐category				*p* < 0.0001		*p* < 0.0001		
N0	8.344	3.119	37.4%		7.4 [4.1‐11.9]		1	
N+	1.098	504	45.9%		9.1 [5.4‐13.9]		0.831	*p* < 0.0001
Grading				*p* < 0.0001		*p* < 0.0001		
High Grade	2.177	741	34.0%		7.3 [4.0‐11.3]		1	
Median Grade	4.564	1.855	40.6%		7.4 [4.2‐11.8]		−0.091	*p* = 0.607
Low Grade	2.701	1.115	41.3%		8.5 [4.6‐13.4]		0.168	

Patients with EAC lost a median of 10.7 years of their expected lifespan, those with CAC 8.5 years and those with GAC 6.8 years. In the multivariate analysis, patients with CAC had a comparable rate of YLL as those with EAC, patients with GAC had a significantly lower rate (Table 3). The other factors such as sex, age and tumor characteristics also showed significant differences in the number of YLL (Table 3).

## 4 Discussion

In the current study, we were able to confirm the results of an earlier research project [20] with a small number of patients (*n* = 214) in a very large number of patients (*n* = 9442) with pT1 adenocarcinoma in the UGI‐tract. These patients lived a median of 7.5 years less than the statistically calculated life expectancy in Germany.

However, it was also shown that these tumors differ significantly depending on their origin topography—esophagus, cardia, or stomach. This applies not only to demographic and tumor‐related factors but also to prognosis and YLL.

Based on the presented data, it is difficult to classify cardiac carcinoma as a tumor of the esophagus or stomach. Even though adenocarcinoma in the UGI‐tract is a tumor that primarily affects men, the proportion of women differs significantly between the locations: esophagus 15%, cardia 19.4%, and stomach 39%. The same applies to the age of patients at diagnosis.

The literature provides varying results on whether cardiac carcinoma belongs to the stomach or the esophagus. Suh et al. analyzed 4524 patients with primary AC of the cardia (*n* = 497) or stomach (*n* = 4027) who underwent surgery at Seoul National Hospital and found no differences in clinicopathological factors [24]. A study from Japan involving 395 patients with CAC used the Siewert classification to answer this question. Based on the results, the authors also conclude that Siewert type II tumors show similar characteristics to gastric carcinoma [25]. Similar results have also been reported from China [26]. All these studies have in common that Siewert Type I is not represented at all or only in small numbers.

The Siewert‐classification is a classification of adenocarcinomas that develop in the transition between the esophagus and the stomach with the aim of defining surgical treatment for these tumors [11]. It describes adenocarcinomas located in an area 5 cm orally to 5 cm aborally of the anatomical gastroesophageal junction. This group of carcinomas contains at least three different entities: Type I: AC of the distal esophagus, whose center is located 1–5 cm above the Z line. Type II: the actual AC of the cardia, (+1 to −2 cm) of the anatomical gastroesophageal junction (muscular junction). Type III: gastric carcinoma arising immediately subcardially, infiltrating the cardia (−2 to −5 cm) from the aboral side. The Siewert‐classification was a response to the increase in AC in the UGI‐tract in Western industrialized nations and the associated challenge in treating patients. Chandrasoma et al. compare EAC with CAC under various aspects and conclude that cardia carcinomas behave like esophageal tumors [27]. Further studies on the Siewert classification from Western industrialized nations show comparable frequencies of the individual types with a large number of type I carcinomas [28, 29, 30].

The numerous and varied publications on the classification of cardiac carcinomas according to their location have also influenced the UICC‐TNM classification of Malignant Tumors over the last 20 years and continue to do so today. The 7th edition [15] compared to the 6th edition, no longer assigns carcinomas diagnosed in the cardia (ICD‐O‐3 C16.0) to the stomach but to the esophagus. In the 8th and actual in the 9th edition of the UICC‐TNM classification system [16, 17] tumors located in the cardia were more differentiated according to the Siewert‐classification. Now a tumor the epicenter of which is within 2 cm of the esophagogastric junction and also extends into the esophagus is classified and staged using the esophageal scheme. Cancers whose epicenter is within the proximal 2 cm of the cardia (Siewert types I/II) are to be staged as esophageal cancers. The changes in these editions from the seventh edition are based on recommendations from the International Gastric Cancer Association Staging Project [31].

When considering the results of our study and the publications listed here, it must be taken into account that the current analysis compares adenocarcinomas of the esophagus (Siewert type I) and the cardia (Siewert types II and III), as the ICD code C16.0 was used for tumor documentation. Another difference from most analyses is that only early‐stage carcinomas were included in the study. Here, it can be assumed that the origin of the tumor and thus its location can be determined more accurately than in advanced tumors. Furthermore, only patients with a histologically confirmed diagnosis of pT1a or pT1b were included. According to the guidelines, the treatment options for these tumors are either endoscopic or surgical removal. In Germany, Siewert type I and II carcinomas are treated in the guidelines for esophageal carcinoma and type III in the guidelines for gastric carcinoma [32]. Unfortunately, information on the treatment used is not available in the cancer registry data set and could therefore not be included in the analysis.

Another difference to other publications may arise from the fact that only precisely defined tumors described histologically as adenocarcinoma were included, that is, no signet ring cell carcinomas or tumors with other types of histology.

The age differences between patients with different tumor locations understandably affect the prognosis and also the loss of life expectancy due to this disease. It is particularly interesting to note that, considering the factors analyzed, patients with CAC have a significantly poorer prognosis compared to the other two locations (Figure 2). This result indicates that patients with pT1 AC in the cardia have a lower survival probability than those with early‐stage cancer in the stomach or esophagus, even when sex, age, depth of tumor infiltration, lymph node involvement, and grading are the same. There may be many reasons for this. One influencing factor could be the choice of therapy, which is not considered in our analysis. While clear treatment procedures are specified in the guidelines for tumors in the esophagus or stomach, there are various options both in practice and in the guidelines. On the other hand, AC in the cardia can have complicated lymphatic drainage pathways, which increase the risk of LNM not being detected either diagnostically or by the therapy performed.

Years of life lost (YLL) are playing an increasingly important role in health research and for individuals. We were able to show that patients who develop AC in the UGI‐tract at a young age have a high risk of dying despite early diagnosis and consequently lose many years of life compared to the statistically expected life expectancy. However, we were also able to show that the group of patients older than 70 years lives on average about 6 years less than the expected lifespan.

There can be many reasons for the reduced life expectancy of these patients. All of these patients received treatment—either surgery or an endoscopic procedure. In every case, the patients' lives changed, whether due to the cancer diagnosis, the consequences of the surgical procedure, or the repeated follow‐up appointments with the fear of a possible recurrence or possible recurrence surgery. Various authors describe the influence of readmission after esophagectomy or gastrectomy on prognosis [33]. In any case, there are opportunities to support patients and possibly reduce YLL. Furthermore, the risk factors for these diseases also appear to have an influence on shorter life expectancy.

One advantage of this analysis is the large number of patients available for evaluation. This made it possible to select cases that had as few additional prognosis‐relevant characteristics as possible. Furthermore, they reflect the reality of healthcare provision in a country, in contrast to evaluations from specialized clinics.

On the other hand, the data come from different sources. The diagnostic procedures alone can cover a broad spectrum, as can the decision on therapy and histopathological assessment.

The ability to use data collected nationwide and made available centrally makes it possible to analyze large cohorts. The data sets were checked for plausibility at ZfKD. Nevertheless, as with other registry data, transmission errors may have occurred, which will be less relevant due to the large number of cases for each subgroup. It cannot be ruled out that patients with this disease are not reported to the tumor registry. This means that there is a certain degree of selection, but this cannot be specified.

The consistency of the results for the number of YLL for the different age groups in the present analysis with the evaluation of a smaller number of patients but very well controlled data [20] reinforces the validity of the statements in this study.

## 5 Conclusion

Patients with AC originating in the transition area between the esophagus and stomach differ from those with AC of the esophagus as well as from those with gastric cancer. It therefore makes sense for carcinomas in the cardia to be given a separate classification and not to be labeled as gastric or esophageal carcinomas. The loss of potential years of life is relevant for patients with AC in the esophagus, cardia, or stomach. Whether the chosen therapy is the cause of this should be the subject of future studies.

## Author Contributions

**Elfriede Bollschweiler:** conceptualization (lead), formal analysis (equal), project administration (equal), writing – original draft (equal). **Arnulf H. Hölscher:** conceptualization (supporting), formal analysis (equal), methodology (equal), project administration (equal), writing – original draft (equal). **Brigitte Schumacher:** data curation (supporting), validation (supporting), writing – review and editing (supporting). **Oliver Pech:** data curation (equal), validation (equal). **Uta Drebber:** data curation (equal), validation (equal).

## Funding

The authors have nothing to report.

## Disclosure

The data used here was provided by the Center for Cancer Data at the Robert Koch Institute (ZfKD) in accordance with the research topic. The content of this data set was checked for plausibility there. In accordance with the data protection regulations of the ZfKD, Elfriede Bollschweiler has sole permission to evaluate this data for the purpose of the survey. The selection of the ICD codes used for the histological type was made in close collaboration with Uta Drebber. All other selections, such as assignments to location and TNM selection, were made from a surgical perspective in collaboration with Arnulf H. Hölscher and, for endoscopy, with Brigitte Schumacher and Oliver Pech. The question of which analyses should be carried out was decided jointly by all authors. All analyses were performed by Elfriede Bollschweiler. The results were primarily checked for plausibility together with Arnulf H. Hölscher. A manuscript was prepared by Elfriede Bollschweiler and read and discussed by all authors.

## Conflicts of Interest

The authors declare no conflicts of interest.

## Data Availability

The data used here was provided by the Center for Cancer Data at the Robert Koch Institute (ZfKD) in accordance with the research topic. The content of this data set was checked for plausibility there. In accordance with the data protection regulations of the ZfKD, Elfriede Bollschweiler has sole permission to evaluate this data for the purpose of the survey.
